# Ripa-56 protects retinal ganglion cells in glutamate-induced retinal excitotoxic model of glaucoma

**DOI:** 10.1038/s41598-024-54075-z

**Published:** 2024-02-15

**Authors:** Lemeng Feng, Shirui Dai, Cheng Zhang, Wulong Zhang, Weiming Zhu, Chao Wang, Ye He, Weitao Song

**Affiliations:** 1 National Clinical Research Center for Geriatric Diseases, Xiangya Hospital of Central South University, No. 87 Xiangya Road, Changsha, Hunan 410008 People’s Republic of China; 2 Eye Center of Xiangya Hospital, Central South University, Changsha, Hunan 410008 People’s Republic of China; 3 Hunan Key Laboratory of Ophthalmology, Changsha, Hunan 410008 People’s Republic of China; 4 Department of Ophthalmology, The Second Xiangya Hospital, Central South University, Changsha, Hunan 410011 People’s Republic of China; 5 Hunan Clinical Research Center of Ophthalmic Disease, Changsha, Hunan 410011 People’s Republic of China

**Keywords:** Glaucoma, Glutamate, Ferroptosis, Apoptosis, Necroptosis, Ripa-56, Cell death in the nervous system, Retina, Drug discovery

## Abstract

Glaucoma is a prevalent cause of blindness globally, characterized by the progressive degeneration of retinal ganglion cells (RGCs). Among various factors, glutamate excitotoxicity stands out as a significant contributor of RGCs loss in glaucoma. Our study focused on Ripa-56 and its protective effect against NMDA-induced retinal damage in mice, aiming to delve into the potential underlying mechanism. The R28 cells were categorized into four groups: glutamate (Glu), Glu + Ripa-56, Ripa-56 and Control group. After 24 h of treatment, cell death was assessed by PI / Hoechst staining. Mitochondrial membrane potential changes, apoptosis and reactive oxygen species (ROS) production were analyzed using flow cytometry. The alterations in the expression of RIP-1, p-MLKL, Bcl-2, BAX, Caspase-3, Gpx4 and SLC7A11 were examined using western blot analysis. C57BL/6j mice were randomly divided into NMDA, NMDA + Ripa-56, Ripa-56 and control groups. Histological changes in the retina were evaluated using hematoxylin and eosin (H&E) staining. RGCs survival and the protein expression changes of RIP-1, Caspase-3, Bcl-2, Gpx4 and SLC7A11 were observed using immunofluorescence. Ripa-56 exhibited a significant reduction in the levels of RIP-1, p-MLKL, Caspase-3, and BAX induced by glutamate, while promoting the expression of Bcl-2, Gpx-4, and SLC7A1 in the Ripa-56-treated group. In our study, using an NMDA-induced normal tension glaucoma mice model, we employed immunofluorescence and H&E staining to observe that Ripa-56 treatment effectively ameliorated retinal ganglion cell loss, mitigating the decrease in retinal ganglion cell layer and bipolar cell layer thickness caused by NMDA. In this study, we have observed that Ripa-56 possesses remarkable anti- necroptotic, anti-apoptotic and anti-ferroptosis properties. It demonstrates the ability to combat not only glutamate-induced excitotoxicity in R28 cells, but also NMDA-induced retinal excitotoxicity in mice. Therefore, Ripa-56 could be used as a potential retinal protective agent.

## Introduction

Glaucoma, a leading cause of irreversible vision loss worldwide, is characterized by the progressive degeneration of retinal ganglion cells (RGCs)^1^. Elevated intraocular pressure (IOP) is one of the most pivotal risk factors of glaucoma. Controlling IOP is still the only effective method for glaucoma treatment^2,3^. However, many patients, especially asians, experience persistent progression of visual field defects despite their IOP are normal, indicating the involvement of other factors^4^. Various mechanisms contribute to RGCs death in glaucoma, such as oxidative stress^5,6^, ocular hypoperfusion^7^, genetics^8^, calpain hyperactivation^9^, glutamate acid excitotoxicity^10^, and neuroinflammation^11^. Unfortunately, there is no effective clinical treatment targeting these non-IOP factors.

Glutamate, a major excitatory neurotransmitter in the central nervous system, plays a vital role in neuronal communication^12^. However, the overactivation of glutamate-gated membrane channels can lead to irreversible neurons damage^13^. This process, known as excitotoxicity, involves the overactivation of NMDA receptors, resulting in calcium influx. Consequently, multiple cell death pathways are triggered in the retina, including calpain activation, oxidative stress, and endoplasmic reticulum stress^14,15^. Given the role of glutamatergic excitotoxicity in glaucoma, inhibiting this process has emerged as a potential target for preventing and treating the condition. Research focused on finding drugs that can slow down this excitotoxicity represents a promising direction for investigating treatments for normal tension glaucoma.

RGCs death is a common consequence in all kinds of glaucoma^1^. Different forms of cell death, such as apoptosis, necrosis, autophagy, ferroptosis and cell pyroptosis, have been thought to be associated with the loss of glaucomatous RGCs^16–21^. Despite efforts to develop potential drugs targeting specific death mechanisms, they have not proven effective in preventing RGCs loss clinically. To address this challenge, it becomes necessary to find drugs that can effectively act on multiple death mechanisms simultaneously.

Ripa-56, a new kind of receptor-interacting protein kinase (RIPK) 1 inhibitor that has been found to be more potent and devoid of off-target effects compared to Necrostatin-1 (Nec-1). Additionally, Ripa-56 has shown promising results in reducing the severity of liver damage^22^. Previous research has demonstrated that RIP-1 inhibitors can prevent glutamate-induced cell death in mouse hippocampal neuron (HT-22) cells^23,24^. The optic nerve is the extracerebral continuation of the central nervous system. Both retinal ganglion cells and HT-22 cells have been used to study glutamate-induced cytotoxicity^25^. We therefore believe that Ripa-56 could be a potential candidate for treating glutamate excitotoxicity. However, there is a lack of relevant research on Ripa-56's effects on glutamate-induced RGC damage. Therefore, this study aims to investigate the protective effects of Ripa-56 in a glutamate-induced excitotoxicity model using the R28 cells line and a NMDA mice model.

## Materials and methods

### Chemicals

Dulbecco's modified Eagle's medium (DMEM) was purchased from Procell (Wuhan, China). Fetal bovine serum (FCS500) was purchased from ExCell Bio (Shanghai, China). Cell counting kit-8 (CCK-8) and Ripa-56 (T7795, 100.00%) were purchased from Topscience (Shanghai, China). Annexin V‐FITC/PI apoptosis detection kit, JC-1 mitochondrial membrane potential assay kit and Hoechst/PI staining buffer were from Beyotime (Shanghai, China). BCA protein assay kit (C05-02001) was purchased from Bioss (Beijing, China). β-actin antibody (66009-1-Ig, 1:20,000) were from Sigma-Aldrich (St. Louis, MO, USA). Anti-Caspase 3 (ab184787), Anti-Bax (ab32503), Anti-Bcl-2 (ab196495), Anti-MLKL (ab243142), p- Anti-MLKL (ab196436), Anti-Brn3a (ab245230, 1:100), Anti-Gpx-4 (ab125066) and Goat Anti-Rabbit IgG H&L (Alexa Fluor 488, ab150077) were obtained from Abcam (Cambridge, UK). Anti-RIP1(#3493S) was purchased from Cell Signaling Technology (CST, Massachusetts, America). Anti-SLC7A11 Polyclonal Antibody (PA1-16893) was from Thermofisher. Anti-IL-6 (EM1701-45, 1:1000) was purchased from HUABIO (Hangzhou, China). Anti RBPMS was from Proteintech (Chicago, America). Goat Anti-Mouse IgG H&L (550017) and Goat Anti-Rabbit IgG H&L (550018) were from Zenbio (China, Chengdu).

### Animals

All experimental procedures were approved by the Ethics Committee of Xiangya Hospital (Central South University, Changsha, Hunan, China), (license No. 202209019, 09/09/2022). The experiments were conducted on C57 BL/6J male mice aged 8–10 weeks and weighing between 22 and 25 g. All the animals were obtained from Hunan SJA Laboratory Animal Co., Ltd (Changsha, Hunan, China) (license No. SYXK (Xiang) 2020-0019). Before the experiments, the mice were adaptively fed for one week under specific pathogen-free feeding (SPF) conditions of a 12-h cycle of light and dark at a temperature of 21 ± 1 ℃. Food and water were available ad libitum. All experiments were conducted in accordance with the guidelines outlined in the Association for Research in Vision and Ophthalmology (ARVO) Statement. The study was carried out in compliance with the ARRIVE guidelines.

### NMDA-induced retinopathy mouse model

We randomly divided the mice into six groups: NMDA group (40 mM), Ripa-56 group (60, 80, 100 μM), NMDA + 60 μM Ripa-56 group, NMDA + 80 μM Ripa-56 group, NMDA + 100 μM Ripa-56 group and control group. The mice were anesthetized with pentobarbital intraperitoneally (1%, 80 mg/kg, intraperitoneal injection). Refer to the previous studies^26–28^, we used oxybrucaine hydrochloride (Shentian Pharmaceutical Co., Ltd.) eyedrop per eye for ocular surface anesthesia to assist pentobarbital to alleviate intraoperative and postoperative ocular surface pain. And use the tropicamide phenylephrine (Shentian Pharmaceutical Co., Ltd.) eyedrop for pupil dilation. Refer to the previous studies^13,28^, we use a 34G needle to make an incision 1 mm posterior to the temporal corneoscleral limbus. For each mouse, we injected 1 µL of fluid into the vitreous cavity of the left eye using microsyringe (Hamilton, Reno, NV, USA), while the right eye remained untreated. All the procedures were conducted under a stereomicroscope. All animals were euthanized three days post intravitreal injection. Fifteen mice intended for HE staining, paraffin section staining, and f-VEP testing were euthanized via cervical dislocation. Twenty-four mice designated for RGCs counting were anesthetized intraperitoneally using pentobarbital (1%, 80 mg/kg). About ten minutes later, the mice were in a fully anesthetized state. When lying supine, their heartbeat and breathing were regular, muscles were relaxed, limbs showed no movement, whisker response was absent, and the pedal reflex disappeared. At this point, we euthanized them by perfusing with 10 ml of physiological saline. The eyeballs were then isolated for further experimentation.

### Histological analysis

The eyeballs were fixed in 4% formalin, dehydrated in a graded series of ethanol, and embedded in paraffin. Subsequently, the eyeballs were cut into 3 µm vertical sections. The slices were stained with hematoxylin and eosin (H&E) and visualized using a light microscope and analyzed using CaseViewer software (3DHISTEC; Sysmex, Switzerland). The thickness of the retinal ganglion cell body complex (GCC) was measured at 600, 1200 and 1800 μm from the optic nerve center.

### Immunofluorescence assay and RGCs counting

The eyeballs were fixed in 4% paraformaldehyde for 30 min, after which the retinas were dissected. Separated vitreous and cut the retina into four pieces, resembling a four-leaf clover. Blocked the retina using 2% BSA dissolved in PBS containing 0.5% Triton-X 100. 1 h later, incubated the retina with anti-Brn3a antibody (1:100) and refrigerated overnight at 4 °C. The next day, washed the retina with 0.5% Triton-X10 three times for at least 5 min. Using 4% paraformaldehyde to fix the retina for 10 min. Then the retina was incubated in the secondary antibody at room temperature for 90 min. After that, washed the retina with PBS and placed it on glass slides and photographed it using a fluorescence microscope. Image-J software was then used to analyzed the immunoreactivity and RGCs number of the sections. The relative RGC density in each group was calculated as a percentage of the mean using the control sample as reference. Statistical analyses of the relative positive cells from each group (in the center areas in each of the four quadrants of the retina) were performed using GraphPad Prism software (version 8.0).

### Immunofluorescence staining of paraffin section

Using 3 µm thick paraffin sections of the eyeballs for staining. Deparaffinized the slices using xylene and alcohol series. Then blocked the endogenous peroxidase activity with 3% hydrogen peroxide. 5 min later, the sections were immersed in antigen repair buffer (0.01 M, pH 6.0) for antigen retrieval using a microwave oven. After cooling, the slices were incubated with the primary antibody at 4 °C. The next day, incubated the slices with a fluorescently-labeled secondary antibody for 1 h at room temperature. DAPI staining buffer was used to tag the nuclei.

### Cell culture

Rat retinal precursor (R28) cells were provided by Central South University. In previous studies, this R28 cell line has been widely used to explore the neuroprotection and pathological mechanism of RGCs in vitro^13,28,29^. The R28 immortalized retinal precursor cell line were maintained in Dulbecco's modified Eagle's medium (DMEM) (Procell, Wuhan, China) containing 1 g/l glucose, and supplemented with 10% FBS (Gibco, Grand Island, NY, USA) at 37 °C with 5% CO_2_. R28 Cells were maintained in T25 culture flasks, and passaging was performed when the cell density reached 80%.

### Cell viability

Seeded R28 cells into 96-well culture plates (5 × 10^3^ cells/well). Then divided it into several groups and treated for 24 h, including glutamate group with concentrations ranging from 0 to 18 mM. Incubated the cells with fresh culture medium containing 10% CCK-8 solution for 2 h at 37 °C. Cell viability was analyzed at 450 nm with a Synergy LX multi-detection microplate reader. Seeded the R28 cells in 96-well plates. Divided it into control group, glutamate group (10 mM) and Ripa-56 group (10 mM glutamate + 0.5–16 μM Ripa-56). After 24 h of intervention, cell viability was assessed using the CCK-8 assay kit. Then divided the cells into several groups and treated for 24 h: 64 μM Ripa-56 group independently. 24 h later, determined cell viability using CCK-8 assay kit.

### Intracellular reactive oxygen species measurement

Using DCF-DA fluorescent probe to detect the levels of intracellular reactive oxygen species (ROS). R28 cells were seeded into 12-well plates (1.5 × 10^5^ cells/well). Added drugs and cultured R28 cells for 24 h. Then incubated the cells with DCF-DA for 30 min at 37 °C. After the incubation, the cells were collected for analysis. The mean fluorescence intensity (MFI) of ROS was then detected using flow cytometry (BD Biosciences, San Jose, CA), and analyzed using FlowJo software.

### Detection of cell apoptosis

Using the Hoechst/PI staining buffer and Annexin V-FITC/PI kit to detect apoptosis in R28 cells. Photographed the cells under the fluorescence microscope or detected it using flow cytometry. FACS was used to differentiate the percentage of cells.

### Detection of mitochondrial membrane potential (Δψ)

Using JC-1 mitochondrial membrane potential assay kit to evaluate the mitochondrial membrane potential (ΔΨm). In brief, incubated R28 cells with drugs for 24 h. Then incubated it with JC-1 working solution at 37 ℃ for 20 min away from light. Later, washed the cells. Then, analyzed it using flow cytometry. The green (JC-1 monomer) and red (JC-1 aggregate) fluorescence were detected with FITC (488 nm) and PE (585 nm) channels respectively. Using FlowJo software to make quantitative analysis.

### Western blotting assay

RIPA lysis buffer (Beyotime, Nanjing, China) was used to extract proteins from R28 cells. Using BCA Protein Assay Kit (Thermo Fisher Scientific, USA) to quantified the protein concentration. Loaded the protein onto SDS-PAGE and then transferred to PVDF membranes. Subsequently, incubated the membrane using blocking buffer at room temperature. Next, cut the membranes into several blots and incubated it with Anti-Caspase 3 (Abcam, Cambridge, UK, ab184787, 1:2000), Anti-Bax (Abcam, Cambridge, UK, ab32503, 1:5000), Anti-Bcl-2 (Abcam, Cambridge, UK, ab196495, 1:2000), Anti-MLKL (Abcam, Cambridge, UK, ab243142, 1:1000), p-Anti-MLKL (Abcam, Cambridge, UK, ab196436, 1:1000), Anti-Gpx-4 (Abcam, Cambridge, UK, ab125066, 1:1000), Anti-RIP1 (CST, Massachusetts, America, #3493S, 1:1000), Anti-SLC7A11 Polyclonal Antibody (Thermofisher, Waltham, MA, PA1-16893, 1:1000) and β-actin antibody (Sigma-Aldrich, St. Louis, MO, USA, 66009-1-Ig, 1:20,000) at 4 °C overnight. The next day, washed the membranes and incubated it in Goat Anti-Mouse IgG H&L (Zenbio, China, 550017, 1:5000) or Goat Anti-Rabbit IgG H&L (Zenbio, China, 550018, 1:5000) for 90 min. The bands signals were detected using an enhanced chemiluminescence reagent (Bio-Rad) and analyzed using the ImageJ software.

### Statistical analysis

Statistical analysis was performed using the GraphPad Prism software (version 8.0). All data are expressed as the means ± SD for at least three independent experiments, and we repeated at least three independent experiments. Multiple data were analyzed using one-way analysis of variance (ANOVA), followed by Tukey’s multiple comparison test. P ≤ 0.05 was regarded as a threshold for significance.

### Ethics approval and consent to patient

All animal experiments were approved by the Ethics Committee of Xiangya Hospital (Central South University, Changsha, Hunan, China). All animal procedures were carried out in accordance with the Guide for the Care and Use of Laboratory Animals published by the US National Institutes of Health.

## Results

### Ripa-56 protects mice from NMDA-induced retinal injury

**Figure 1:**
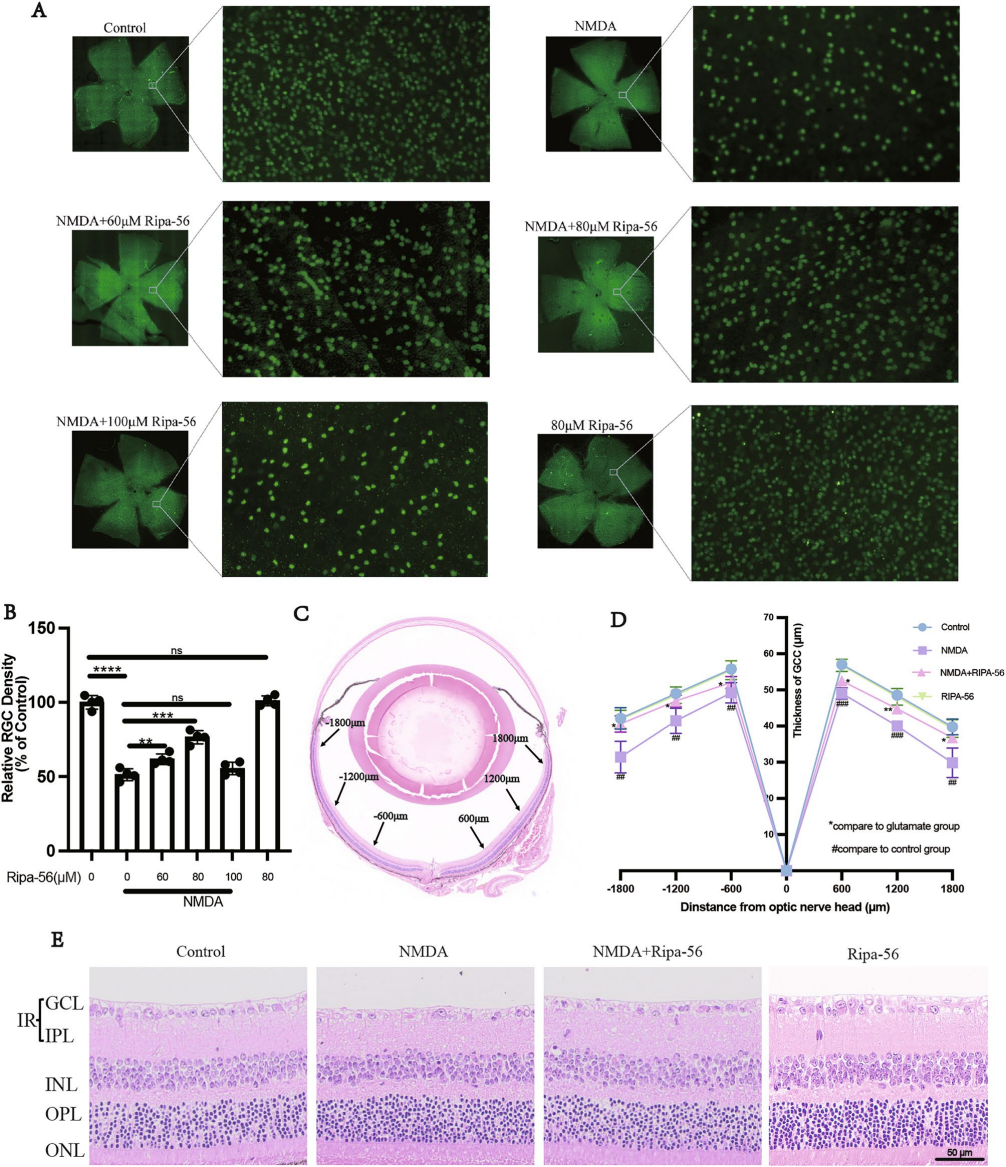
Protective effect of Ripa-56 in NMDA-induced mice retinal injury model. **(A)** Effects of Ripa-56 on NMDA-induced RGCs injury in mice model (n = 4). NMDA and Ripa-56 were injected into the vitreous cavity. 3 days later, we made retinal slices. RGCs were fluorescently labeled with Brn3a antibody. (**B)** Relative RGCs density in Control group (100 ± 4.57%), NMDA group (51.36 ± 4.00%), NMDA + 60 µM Ripa-56 group (61.76 ± 3.54%), NMDA + 80 µM Ripa-56 group (76.52 ± 4.41%), NMDA + 100 µM Ripa-56 group (55.49 ± 4.19%), 80 µM Ripa-56 group (101.02 ± 3.33%) (n = 4). **(C,D)** Thickness of the retinal ganglion cell body complex (GCC) at 600, 1200, and 1800 μm from the optic disc 3 days after intravitreal injection of NMDA and Ripa-56. (**E**) Effects of Ripa-56 on retinal morphology in NMDA model rats. Hematoxylin and eosin staining was performed 3 days after intravitreal injection of NMDA and Ripa-56. INL, inner nuclear layer; ONL, outer nuclear layer; GCL, ganglion cell layer; IPL, inner plexiform layer. IR consists of GCL and IPL. The results were recorded as mean ± SD from at least three independent experiments. Data analyzed via one-way ANOVA and Tukey’s post-test. *p < 0.05,**p < 0.01, ***p < 0.001, ****p < 0.0001, ns p > 0.05. #p < 0.05, ##p < 0.01, ###p < 0.001 , ####p < 0.0001.

Different concentrations of Ripa-56 were set to explore the optimal therapeutic concentration. The distribution of RGCs was quantitatively observed using immunofluorescent staining of Brn3a on retinal slides^30^. The RGCs were dense and evenly distributed in control group. Treatment with 60 µM (61.76 ± 3.54%) or 80 µM (76.52 ± 4.41%) Ripa-56 resulted in increased RGC density compared to the NMDA group (51.36 ± 4.00%), but it remained less than that of the control group (100 ± 4.57%) (p < 0.05). However, when the Ripa-56 concentration was raised to 100 µM, the RGCs density decreased (55.49 ± 4.19%) (p < 0.05), but the difference was not statistically significant when compared to the NMDA group (Fig. 1A, B). Interestingly, when the group was given 60 µM (104.03 ± 3.66%) or 80 µM (99.09 ± 3.08%) Ripa-56 alone, the RGCs was unchanged compared to the control group (100.00 ± 5.58%) (p > 0.05), when the Ripa-56 concentration was raised to 100 µ M, the RGCs density decreased (78.55 ± 5.98%) (p < 0.05) (Supplement Fig. 1).

To further observe retinal structures, H&E staining was perfprmed (Fig. 1C–E). We found that the retinal structures in the control group appeared clear and well-organized. We determined the thickness of the retinal ganglion cell body complex (GCC) at distances 600, 800, and 1600 μm from the optic center of optic disc. The thickness of GCC was found to be thinner at all measurement points in the NMDA group compared with control group (P < 0.05). And this damage induced by NMDA was prevented in the Ripa-56 treated group, but have no changes in Ripa-56 group compared to control group.

### Effect of Ripa-56 on retinal inflammation in NMDA model mice

**Figure 2:**
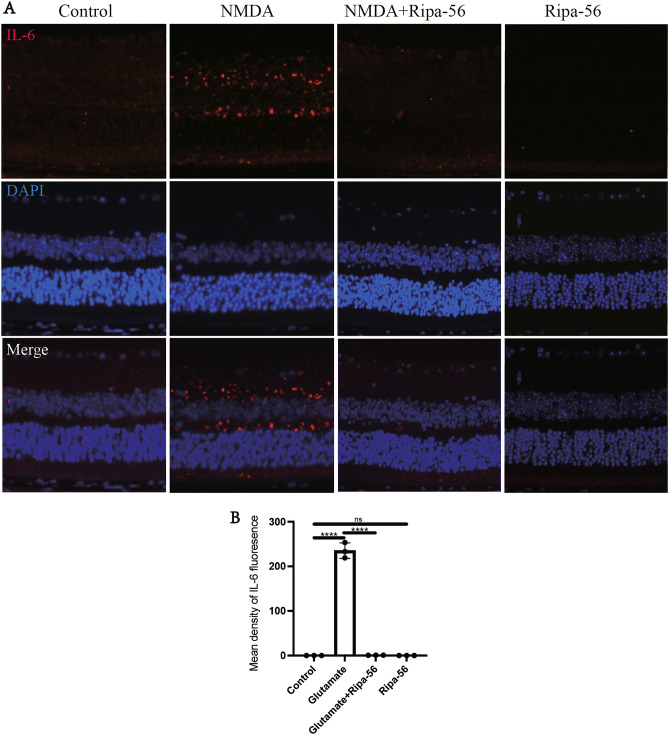
Effect of Ripa-56 on retinal inflammation in NMDA model mice. (**A,B**) Three days after intravitreal injection of NMDA and Ripa-56, paraffin sections were collected and processed for immunofluorescence experiments to measure the fluorescence intensity of IL-6 of the retinal sections in Control group (0.075 ± 0.025), NMDA group (235.23 ± 17.42), NMDA + Ripa-56 group (0.82 ± 0.14) and Ripa-56 group (0.055 ± 0.032) under a fluorescence microscope (n = 3). DAPI was used to label the cell nucleus.

IL-6 has been identified as a pivotal factor in the development of neuroinflammatory diseases^31,32^. In previous studies, it was found that glutamate excitotoxicity triggers inflammation, leading to an increase in proinflammatory factors such as TNF α, IL-1 β and IL-6^33,34^. To visualize IL-6, we performed fluorescent staining of retinal sections^35^. The results indicate that IL-6 expression was significantly higher in the NMDA group (235.23 ± 17.42) compared to control group (0.07 ± 0.03) (P < 0.05), while Ripa-56 treatment (0.82 ± 0.14) resulted in a reduction of IL-6 expression (Fig. 2A, B).

### Ripa-56 relieve the glutamate-induced damage to R28 cell

**Figure 3:**
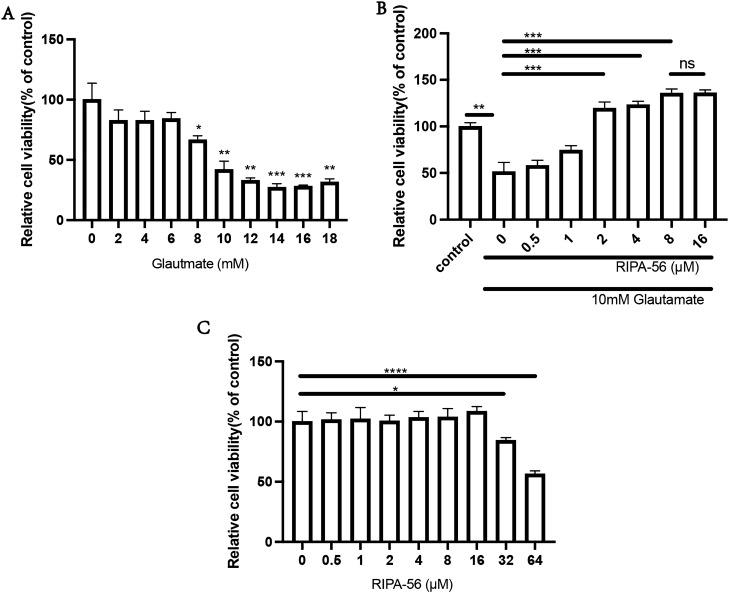
Ripa-56 protect against glutamate induced damage to R28 cell line. (**A**) The effect of 0 mM (100.00 ± 13.64%), 2 mM (82.64 ± 8.75%), 4 mM (82.64 ± 7.62%), 6 mM (84.21 ± 5.11%), 8 mM (66.60 ± 3.46%), 10 mM (41.93 ± 7.01%), 12 mM (32.89 ± 2.20%), 14 mM (27.21 ± 3.11%), 16 mM (28.02 ± 1.10%) and 18 mM (31.57 ± 2.75%) of glutamate on R28 cells (n = 3). (**B**) Protection of Ripa-56 against glutamate-induced excitotoxicity in R28 cells (n = 3). The relative cell viability in control group (100.00 ± 4.03%), 0 µM Ripa-56 group (51.40 ± 10.01%), 0.5 µM Ripa-56 group (58.04 ± 5.67%), 1 µM Ripa-56 group (74.49 ± 4.89%), 2 µM Ripa-56 group (119.47 ± 6.80%), 4 µM Ripa-56 group (123.50 ± 3.87%), 8 µM Ripa-56 group (135.50 ± 4.75%) and 16 µM Ripa-56 group (135.84 ± 3.40%). (**C**) The effect of 0 µM (100.00 ± 8.41%), 0.5 µM (101.30 ± 5.84%), 1 µM (102.04 ± 9.64%), 2 µM (100.48 ± 4.84%), 4 µM (103.34 ± 5.12%), 8 µM (103.69 ± 7.08%), 16 µM (108.38 ± 4.07%) 32 µM (84.35 ± 2.36%) and 64 µM (56.49 ± 2.57%) of Ripa-56 treatment for 24 h on the R28 cell viability (n = 3). The results were recorded as mean ± SD from at least three independent experiments. Data analyzed via one-way ANOVA and Tukey’s post-test. *p < 0.05, **p < 0.01, ***p < 0.001 , ****p < 0.0001, ns p > 0.05.

Using a Cell Counting Kit-8 (CCK-8) to assess the cell viability of R28 cell treated with different concentrations of glutamate for 24 h. The results showed (Fig. 3A) that, compared with the control group (100 ± 13.64%), the cell viability in the glutamate group decreased in a concentration-dependent manner (p < 0.05). Among the different concentrations tested, 10 mM glutamate reduced the cell viability of the R28 cell line by 41.93 ± 7.01%, so we chose 10 mM as the fixed concentration for the later experiment. To investigate the effect of Ripa-56 on cell viability, we treated R28 cells with glutamate and different concentrations of Ripa-56 for 24 h, and assessed the outcomes using a CCK-8 assay kit.

It shows that, compared with the glutamate group, cell viability of R28 was increased after 2 μM (119.47 ± 6.80%) , 4 μM (123.29 ± 3.87%), 8 μM (135.50 ± 4.75%) and 16 μM (135.84 ± 3.40%) Ripa-56 treated (p < 0.05), which suggests that Ripa-56 has a significant protective effect on glutamate induced injury of the R28 cell (Fig. 3B). There was no statistical difference in cell viability between the 8 μM and 16 μM Ripa-56 treatment groups. So, we chose the 2 μM, 4 μM and 8 μM for subsequent experiments. To ensure the safety of Ripa-56, we tested its potential toxicity on R28 cells by treating them with corresponding concentrations of Ripa-56 alone. The results showed that the addition of 0.5 μM (101.30 ± 5.84%), 1 μM (102.04 ± 9.64%), 2 μM (100.48 ± 4.84%), 4 μM (103.34 ± 5.12%), 8 μM (103.69 ± 7.08%) and 16 μM (108.38 ± 4.07%) Ripa-56 had no significant effect on the cell viability of the R28 cell line (Fig. 3C).

### Ripa-56 protects R28 cell line against glutamate-induced apoptosis

**Figure 4:**
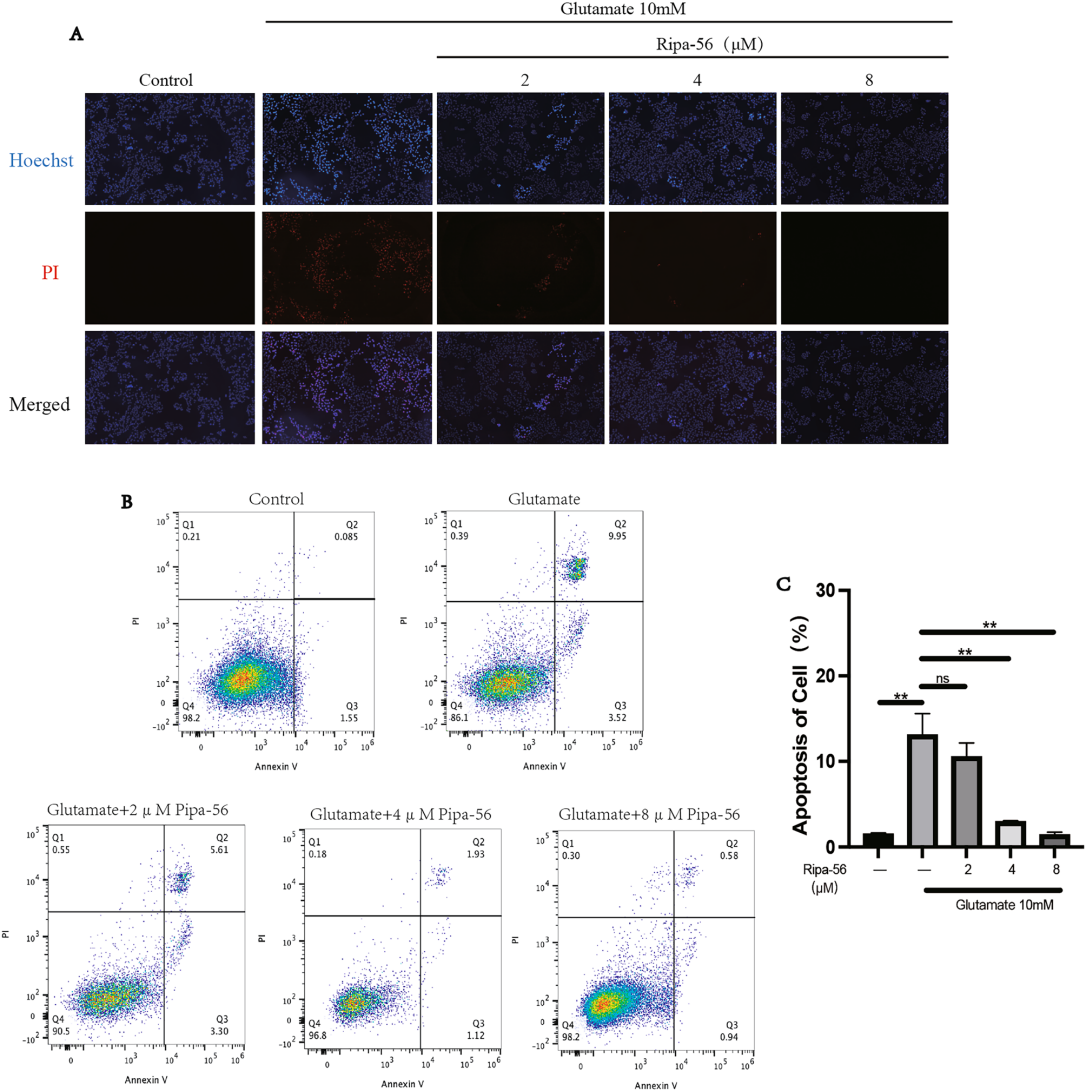
Ripa-56 protect R28 cells against glutamate induced apoptosis. (**A**) The effect of Ripa-56 on apoptosis was observed using Hoechst/PI staining kit and observed under a fluorescence microscope. (**B**) After Annexin-V-FITC/PI staining, detected the effect of Ripa-56 on apoptosis in R28 cells by flow cytometry (n = 3). (**C**) Effects of Ripa-56 on the apoptosis percentage in control group (1.52 ± 0.11%), glutamate group (13.12 ± 2.46%), glutamate + 2 µM Ripa-56 group (10.57 ± 1.59%), glutamate + 4 µM Ripa-56 group (2.95 ± 0.11%) and glutamate + 8 µM Ripa-56 group (1.43 ± 0.30%) (n = 3). The results were recorded as mean ± SD from at least three independent experiments. Data analyzed via one-way ANOVA and Tukey’s post-test. *p < 0.05, **p < 0.01, ***p < 0.001 , ****p < 0.0001, ns p > 0.05.

Neuronal apoptosis can occur during a period of glutamate excitotoxicity^36^. To explore the impact of Ripa-56 on glutamate-induced apoptosis in R28 cells, we employed the Hoechst/PI staining kit (Fig. 4A). The results demonstrated that the cells in control group were a normal shape with no apoptotic cells. In contrast, the cells in the glutamate group showed reduced volume, and the proportion of apoptotic cells was significantly higher compared to the control group. However, Ripa-56 treatment significantly reduced glutamate-induced apoptosis.

To further investigate apoptosis, we used Annexin V/PI and analyzed and analyzed the cells via flow cytometry (Fig. 4B, C). The results indicated that the percentage of apoptosis in the glutamate group (13.12 ± 2.46%) increased significantly compared to the normal group (1.52 ± 0.11%) (p < 0.05). However, 2 μM (10.57 ± 1.59%), 4 μM (2.95 ± 0.11%) and 8 μM (1.43 ± 0.30%) Ripa-56 treatment significantly reduced glutamate-induced apoptosis. This observation aligns with the findings obtained from the Hoechst/PI staining under the microscope.

### Preliminary mechanism of Ripa-56 in ameliorating apoptosis induced by glutamate

**Figure 5:**
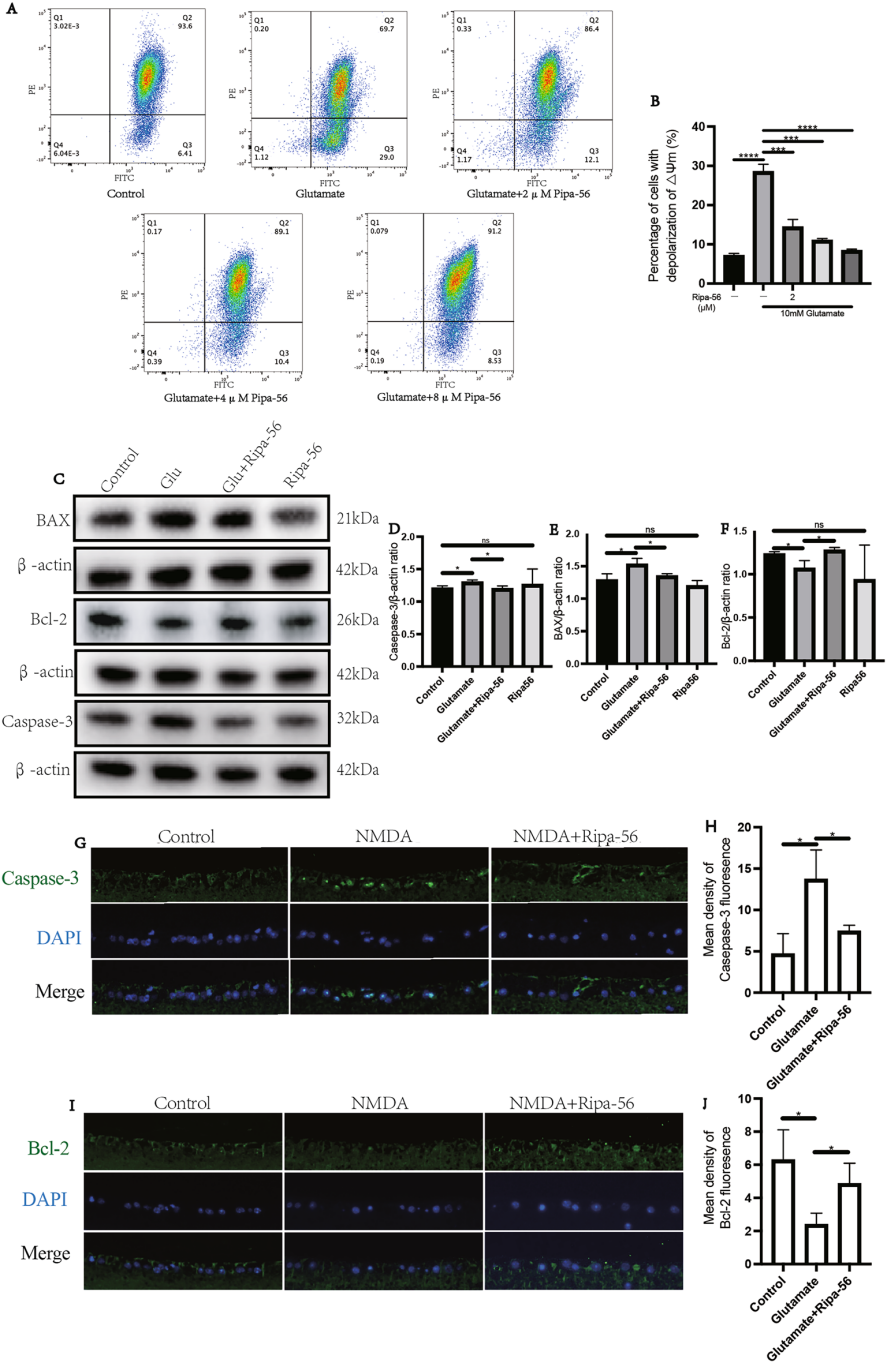
Mechanism of Ripa-56 preventing glutamate-induced apoptosis in R28 cells. (**A**) The effect of Ripa-56 on mitochondrial membrane potential of R28 cell line treated with 10 mM glutamate for 24 h was detected using the flow cytometry after JC-1 staining (n = 3). (**B**) Effect of Ripa-56 on the percentage of mitochondrial membrane potential depolarized R28 cells in control group (7.08 ± 0.58%), glutamate group (28.47 ± 1.96%), glutamate + 2 µM Ripa-56 group (14.37 ± 1.99%), glutamate + 4 µM Ripa-56 group (10.97 ± 0.51%) and glutamate + 8 µM Ripa-56 group (8.40 ± 0.40%) (n = 3). (**C**) Effects of Ripa-56 on Caspase-3, BAX and Bcl-2 protein relative expression (n = 3). (**D**) Relative expression of Caspase-3 protein in control group (1.20 ± 0.042), glutamate group (1.29 ± 0.036), glutamate + Ripa-56 group (1.19 ± 0.047) and Ripa-56 group (1.26 ± 0.24). (**E**) Relative expression of BAX protein in control group (1.28 ± 0.10), glutamate group (1.53 ± 0.10), glutamate + Ripa-56 group (1.35 ± 0.039) and Ripa-56 group (1.19 ± 0.084). (**F**) Relative expression of Bcl-2 protein in control group (1.23 ± 0.030), glutamate group (1.06 ± 0.09), glutamate + Ripa-56 group (1.27 ± 0.036) and Ripa-56 group (0.93 ± 0.40). (**G–J**) Three days after intravitreal injection of NMDA and Ripa-56, paraffin sections were collected and processed for immunofluorescence experiments to measure the fluorescence intensity of Casepase-3 under a fluorescence microscope (**G,H**) in control group (4.67 ± 2.46), glutamate group (13.70 ± 3.55) and glutamate + Ripa-56 group (7.43 ± 0.72), and Bcl-2 (I, J) in control group (6.29 ± 1.82), glutamate group (2.38 ± 0.70) and glutamate + Ripa-56 group (4.86 ± 1.24) (n = 3). The results were recorded as mean ± SD from at least three independent experiments. Data analyzed via one-way ANOVA and Tukey’s post-test. *p < 0.05, **p < 0.01, ***p < 0.001 , ****p < 0.0001, ns p > 0.05.

Mitochondria play a crurial role in triggering apoptosis^37^. This process involves the disruption of the mitochondrial membrane, leading to a decrease in mitochondrial transmembrane potential (ΔΨm)^38,39^. To investigate whether glutamate-induced apoptosis in the R28 cell line occurs through the mitochondrial pathway, we utilized the JC-1 staining kit. The results revealed a significant increase in the percentage of ΔΨ depolarized cells in the R28 cells after 24 h of glutamate exposure (p < 0.05) (Fig. 5A, B). Compared with the glutamate group (28.47 ± 1.96%), the percentage of depolarized cells decreased in a 2 μM (14.37 ± 1.99%), 4 μM (10.97 ± 0.51%) and 8 μM (8.40 ± 0.40%) Ripa-56 group (p < 0.05).

Caspase-3 is a key protein involved in caspase-dependent apoptosis, while Bcl-2 is famous as a key anti-apoptotic protein, and dysregulation of the Bcl-2-associated X protein (BAX) often leads to apoptosis. To explore the anti-apoptotic effect of Ripa-56, we assessed the expression of these apoptosis -related proteins using western blotting (Fig. 5C–F). The results showed that, compared to control group (1.28 ± 0.10), the expression of BAX proteins increased in the glutamate group (1.53 ± 0.10) (p < 0.05). Compared to control group (1.20 ± 0.04), the expression of caspase-3 proteins increased in the glutamate group (1.29 ± 0.03), while the expression of Bcl-2 proteins decreased in the glutamate group (1.06 ± 0.09) compared to control group (1.23 ± 0.03) (p < 0.05). The expression of these proteins in group only added Ripa-56 have no significant difference compared to control group. (P > 0.05) We further examined Caspase-3 and Bcl-2 expression in vivo by immunofluorescence staining of mouse retinal sections (Fig. 5G–J).

The results were consistent with the findings from the cell experiments. Compared to the control group (4.67 ± 2.46), the Caspase-3 fluorescence intensity increased in the NMDA group (13.70 ± 3.55), and the fluorescence intensity of Bcl-2 decreased in NMDA group (2.38 ± 0.70) compared to the control group (6.29 ± 1.82) (p < 0.05). However, after treatment with Ripa-56, the fluorescence intensity of Caspase-3 (7.43 ± 0.72) decreased and Bcl-2 (4.86 ± 1.24) fluorescence intensity increased compared to the NMDA group (p < 0.05).

### Ripa-56 attenuates glutamate-induced necroptosis in a glaucoma model

**Figure 6:**
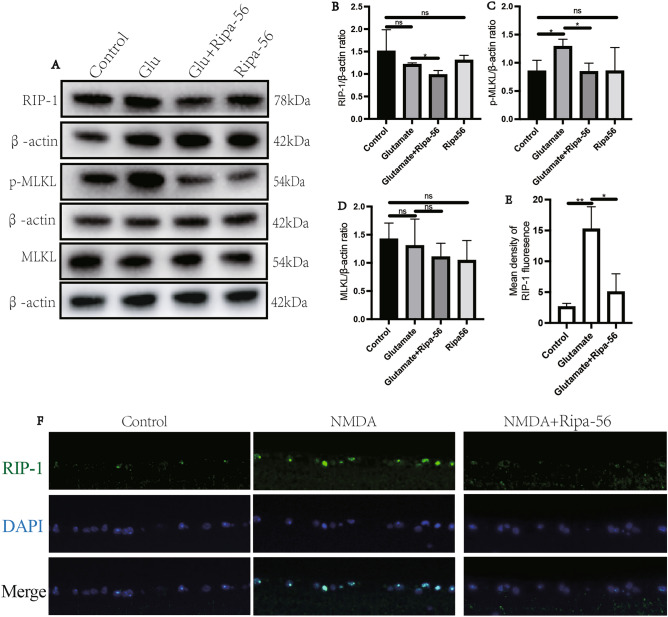
Mechanism of Ripa-56 preventing glutamate-induced apoptosis in R28 cells. (**A**) Effects of Ripa-56 on RIP-1, MLKL and p-MLKL protein relative expression (n = 3). (**B**) Relative expression of RIP-1 protein in control group (1.51 ± 0.48), glutamate group (1.21 ± 0.038), glutamate + Ripa-56 group (0.98 ± 0.098) and Ripa-56 group (1.30 ± 0.11). (**C**) Relative expression of p-MLKL protein in control group (0.85 ± 0.20), glutamate group (1.29 ± 0.13), glutamate + Ripa-56 group (0.84 ± 0.15) and Ripa-56 group (0.85 ± 0.42). (**D**) Relative expression of MLKL protein in control group (1.42 ± 0.29), glutamate group (1.30 ± 0.48), glutamate + Ripa-56 group (1.10 ± 0.24) and Ripa-56 group (1.04 ± 0.35). (**E,F**) Three days after intravitreal injection of NMDA and Ripa-56, paraffin sections were collected and processed for immunofluorescence experiments to measure the fluorescence intensity of RIP-1 of the retinal sections in control group (2.62 ± 0.55), glutamate group (15.22 ± 3.62) and glutamate + Ripa-56 group (5.05 ± 2.92) under a fluorescence microscope (n = 3). The results were recorded as mean ± SD from at least three independent experiments. Data analyzed via one-way ANOVA and Tukey’s post-test. *p < 0.05, **p < 0.01, ***p < 0.001 , ****p < 0.0001, ns p > 0.05.

Ripa-56 was initially identified as an RIP1 inhibitor^22^. Previous research has indicated that necroptosis can be activated in the glutamate excitotoxicity model^28^. RIP1 and MLKL are the main signals involved in necroptosis. To explore the effect of Ripa-56 on these signals, we conducted a western blot assay to examine the expression of RIP-1, p-MLKL, and MLKL proteins (Fig. 6A–D). The results revealed that, compared to glutamate group (1.21 ± 0.04), RIP1 protein expression significantly decreased in the Ripa-56 group (0.98 ± 0.10) (p < 0.05). Compared to glutamate group (1.28 ± 0.13), p-MLKL protein expression significantly decreased in the Ripa-56 group (0.84 ± 0.15) (p < 0.05), while there was no significant difference in MLKL protein expression (p > 0.05). In addition, we performed immunofluorescence experiments to examined RIP1 expression in the retina (Fig. 6E, F). It showed that the fluorescence intensity of RIP1 increased in the mice retina after intravitreal injection of NMDA (15.22 ± 3.62) (p < 0.05). However, the RIP1 fluorescence intensity significantly reduced in the Ripa-56 treated group (5.05 ± 2.92) compared to the NMDA group (p < 0.05).

### Ripa-56 attenuates glutamate-induced ferroptosis in a glaucoma model

**Figure 7:**
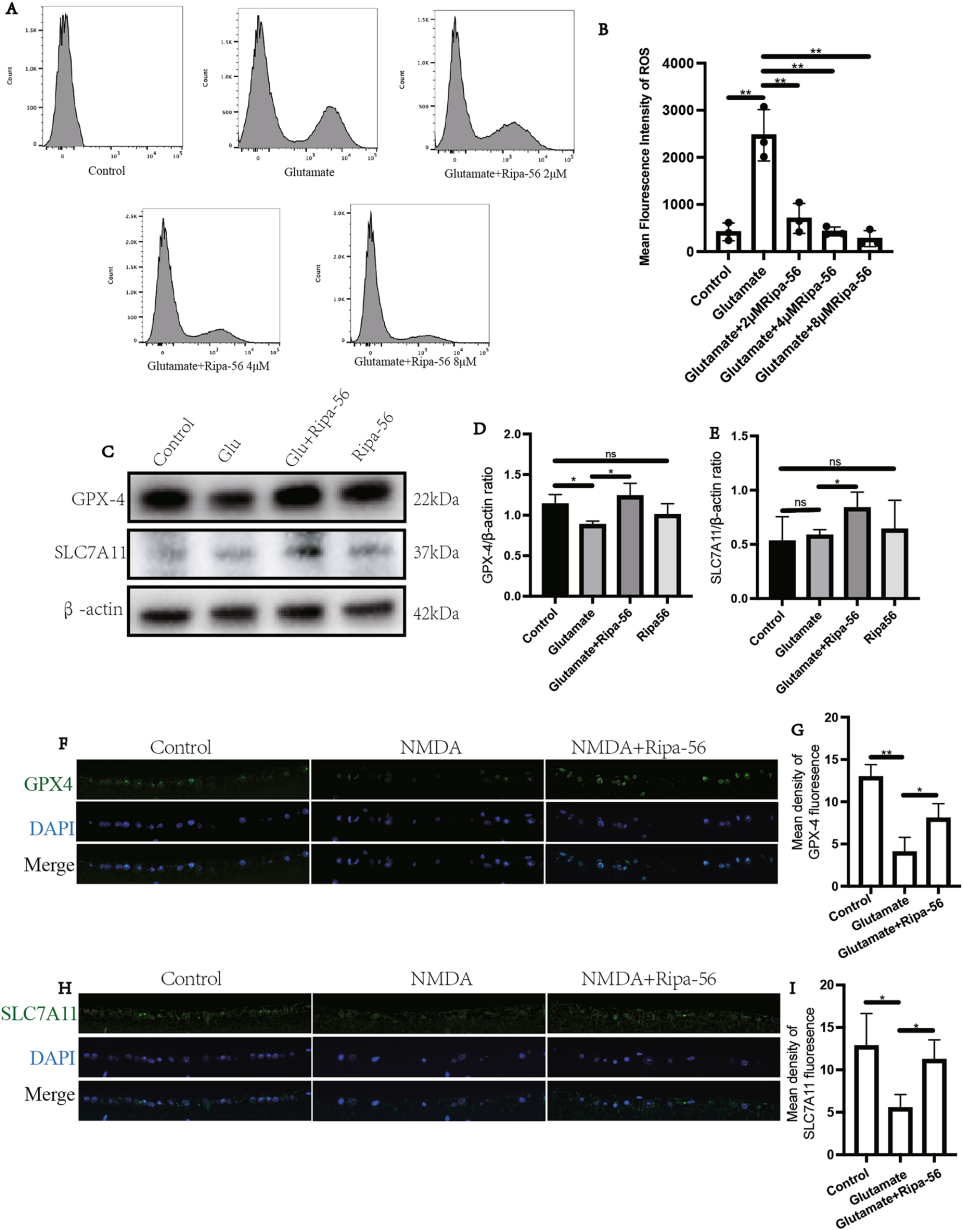
Ripa-56 attenuates glutamate-induced ferroptosis in glaucoma model. (**A,B**) Flow cytometric technique to detect the ROS generation in R28 cells (n = 3). (**C**) Effects of Ripa-56 on GPX-4 and SLC7A11 protein relative expression (n = 3). (**D**) Relative expression of GPX-4 protein in control group (1.13 ± 0.12), glutamate group (0.88 ± 0.048), glutamate + Ripa-56 group (1.23 ± 0.16) and Ripa-56 group (1.00 ± 0.14). (**E**) Relative expression of SLC7A11 protein in control group (0.53 ± 0.23), glutamate group (0.58 ± 0.053), glutamate + Ripa-56 group (0.83 ± 0.15) and Ripa-56 group (0.64 ± 0.27). (**F–H**) Three days after intravitreal injection of NMDA and Ripa-56, paraffin sections were collected and processed for immunofluorescence experiments to measure the fluorescence intensity of GPX-4 (**F,G**) in control group (12.93 ± 1.46), glutamate group (4.03 ± 1.77) and glutamate + Ripa-56 group (8.02 ± 1.74), and SLC7A11 (**H,I**) in control group (12.80 ± 3.84), glutamate group (5.49 ± 1.59) and glutamate + Ripa-56 group (11.20 ± 2.35) under a fluorescence microscope (n = 3). The results were recorded as mean ± SD from at least three independent experiments. Data analyzed via one-way ANOVA and Tukey’s post-test. *p < 0.05, **p < 0.01, ***p < 0.001 , ****p < 0.0001, ns p > 0.05.

We utilized flow cytometric techniques to detect intracellular reactive oxygen species (ROS) levels (Fig. 7A, B). The mean fluorescence intensity (MFI) significantly increased in the glutamate group (2470 ± 544.72) (p < 0.05). However, Ripa-56 treatment ameliorated the ROS elevation induced by glutamate. At a concentration of 2 μM Ripa-56, the ROS MFI decreased to 704.00 ± 316.11 (p < 0.05). Furthermore, when 4 μM Ripa-56 (428.67 ± 92.39) was added, the ROS MFI returned to the level of the normal group (p < 0.05).

Glutamate has been well established as the classical ferroptosis inducer^40^. The SLC7A11/GPX-4 pathway is a classical defense mechanism against ferroptosis, known to regulate intracellular ROS. We assessed the intracellular SLC7A11 and GPX-4 protein expression and found that the Ripa-56 treatment in R28 cells increased the expression of SLC7A11 and GPX-4 proteins. (Fig. 7C–E). It was found that the expression of SLC7A11 protein in the glutamate group (0.58 ± 0.05) had no statistical significance compared with control group (0.53 ± 0.23) (P > 0.05). However, the expression of SLC7A11 was higher after Ripa-56 treatment (0.83 ± 0.15) compared with the glutamate group (P < 0.05). GPX-4 protein expression in the glutamate group (0.88 ± 0.05) was lower than that in the control group (1.13 ± 0.12) (P < 0.05). And the expression of GPX-4 was increased after the treatment of Ripa-56 (1.23 ± 0.16) (P < 0.05). We subsequently examined the expression of SLC7A11 and GPX-4 proteins in paraffin sections of the mouse retina using immunofluorescence (Fig. 7F–I). We observed that the fluorescence intensity of SLC7A11 and GPX-4 proteins was lower in the NMDA group compared to control group (p < 0.05). However, in the Ripa-56 treatment group, the fluorescence intensity of these two proteins were enhanced compared to NMDA group (p < 0.05).

## Discussion

**Figure 8:**
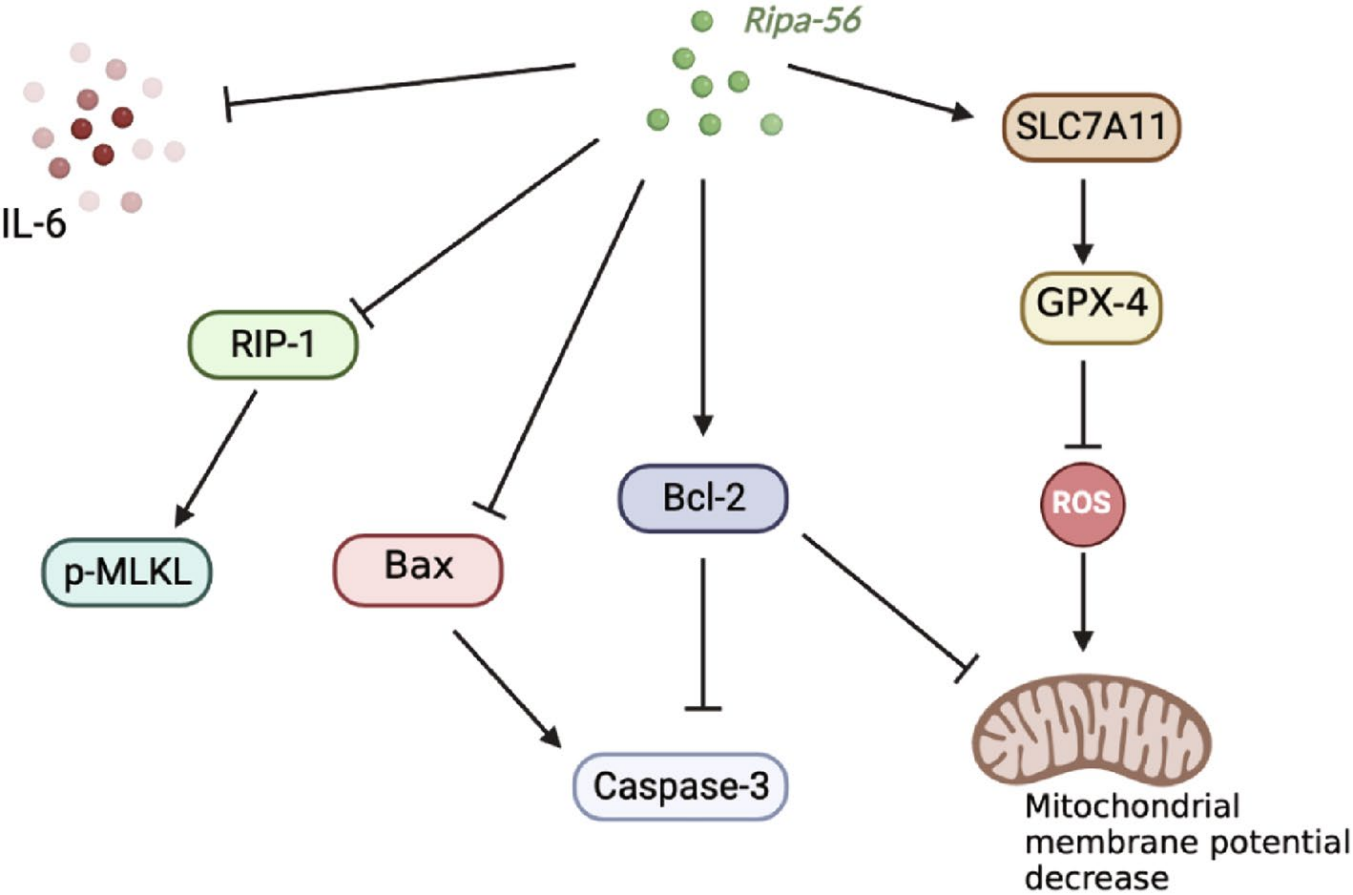
Protective effect of Ripa-56 on RGCs.

Glaucoma, a group of blindness diseases characterized by the progressive degeneration of retinal ganglion cells, remains the leading cause of irreversible vision loss worldwide^1^. Glutamate, an excitatory neurotransmitter, can lead to cell death at high concentrations^41^. For glaucoma patients, excessive activation of NMDA receptors induced by glutamate leads to an excessive Ca^2+^ influx, resulting in ganglion cell excitotoxicity and retina damage^42,43^. Although NMDA receptor antagonists have been considered potential glaucoma drugs to inhibit glutamate excitotoxicity^44–46^. Their practical anti-glaucoma efficacy has not been ideal, as seen with memantine in phase III clinical trials, where it failed to delay glaucoma progression^47^. This prompted us that using NMDA receptors antagonism did not effectively alleviate glaucomatous glutamate excitotoxicity. Recent studies shown that necrostatin-1 protects RGCs in glutamate excitotoxicity models by inhibiting necroptosis^28^. In our study, we discovered that Ripa-56, an RIP1 kinase inhibitor, not only inhibits necroptosis but also alleviates inflammation, ferroptosis, and apoptosis in glutamate excitotoxicity models^22^ (Fig. 8). Ripa-56 can exert protective effects in R28 cell glutamate excitotoxicity models and NMDA mouse models.

To mimic the damage of high glutamate levels on retinal damage, we intravitreally injected a certain concentration of NMDA into mice eyes, aiming to explore the effects of Ripa-56 on NMDA-induced retinal damage. The results showed that three days after intravitreal injection of NMDA, there was a significant reduction in RGCs and a decrease in the thickness of GCC. Ripa-56 partially reversed this damage. In the in vitro study, we induced damage by adding glutamate to R28 cell cultures, and it was found that Ripa-56 treatment increased the cell viability of the R28 cells. Both the in vivo and in vitro results indicate that the Ripa-56 has a protective effect on R28 cells and mice retina injuries caused by glutamatergic excitotoxicity.

Inflammation plays an important role in glaucoma^48^. Previous studies have shown that extrasynaptic glutamate diffusion can trigger neuroinflammation^49^. Some researchers have observed an increase in the level of proinflammatory cytokines, such as TNF-α and IL 6, in the retinas of individuals with glaucoma^50^. This suggests that a potential association between glutamate excitotoxicity and inflammation in glaucoma. McNearney et al. found that glutamate could influence osteoarthritis through IL-6 signaling^51,52^. Additionally, a study on subclinical atherosclerosis demonstrated an independent positive correlation between glutamate and IL-6 levels^53^. Thus, it is important to study the inflammatory effect of glutamate excitotoxicity on the retina and the potential impact of Ripa-56 on this process. In our study, we examined the expression of IL-6 in the retina. The results indicated an increase in IL-6 expression in the NMDA group. However, the Ripa-56 treatment significantly reduced IL-6 expression. Based on these, we hypothesized that Ripa-56 might alleviate glutamate excitotoxicity-induced retinal inflammation in glaucoma model. However, the specific mechanism requires further studied.

The glutamatergic excitotoxicity could induce neuronal and glial apoptosis^54,55^. This was confirmed in our study. Through flow cytometry assays and fluorescent staining, we demonstrated that the glutamate induces apoptosis in R28 cells. Additionally, we found that Ripa-56 had a significant protective effect against glutamate-induced apoptosis. The mitochondria-mediated apoptosis pathway is one of the main pathways of apoptosis. The stabilization of mitochondrial membrane potential (MMP) is essential for maintaining the normal physiological function of mitochondria. Oxidative stress can decrease MMPs, leading to the release of cytochrome c (Cyt-c), initiating a caspases cascade and ultimately causing apoptosis^56,57^. By preventing the reduction of MMPs, apoptosis can be inhibited^58^. MMP is often used as an indicator to assess mitochondrial function as changes in MMP precede mitochondrial lesions. The Bcl-2 family proteins play a vital role in regulating apoptosis^59^. Pro-apoptotic Bax activation triggers MMPs reduction and Cyt-c release, activating Caspase-3 and inducing apoptosis, while anti-apoptotic Bcl-2 acts in the opposite manner. In our study, Ripa-56 reversed the glutamate-induced decrease in MMP, down-regulation Bcl-2 expression, and up-regulation Bax and caspase-3 expression in R28 cells to a certain degree. This suggests that Ripa-56 may inhibit the mitochondrial apoptosis pathway and enhance the anti-apoptotic ability of cells. Our observations in the mouse retina similar to the trends observed in the cell experiments, indicating that Ripa-56 can alleviate the damage to RGCs caused by glutamate excitotoxicity by inhibiting apoptosis.

Necrotic apoptosis is one of the programmed cell death forms associated with neurodegenerative diseases, such as Alzheimer's disease^60–62^. The RIP1/MLKL pathway is the classical regulatory pathway of necroptosis^63^. Glutamate excitotoxicity induces necroptosis in RGCs through activation of the RIP1/MLKL pathway both in vivo and in vitro. The necroptosis inhibitor could increase the survival of RGCs^28^. In our study, western blotting revealed that the Ripa-56 treated group exhibited decreased RIP1 and p-MLKL protein level compared to the glutamate group. Additionally, we observed changes in RIP1expression the retinal ganglion cell layer through immunofluorescence, and the trend of it similar to those in the cell experiments. These findings suggest that Ripa-56 alleviates glutamate-induced necroptosis by inhibiting the RIP1/MLKL pathway.

Ferroptosis is an iron-dependent programmed cell death process. Proteomic analysis by Su et al. indicated that ferroptosis may play an important role in RGCs loss in the NMDA glaucoma model^64^. The SLC7A11/GPX 4 pathway serves as the dominant antioxidant system in cells, protecting against ferroptosis. Reduction of SLC7A11 can induce ferroptosis by affecting GPX-4 activity. SLC7A11 responsible for transporting cystine into the cell, where it is oxidized to cysteine, facilitating GSH synthesis, an essential factor for GPX-4^65,66^. In our study, Ripa-56 inhibited ferroptosis by reducing glutamate excitotoxicity-induced lipid ROS accumulation through the activation of the SLC7A11/GPX 4 signaling pathway. However, it is important to note that the molecular mechanisms upstream of ferroptosis are complex, and the effect of Ripa-56 on other ferroptosis pathways in the glutamate model requires further exploration.

It's worth noting that our study has certain limitations. Firstly, we investigated the neuroprotective effect of Ripa-56 in the glutamate excitotoxicity model on RGCs. However, it's well-known that glutamate excitotoxicity is just one of the causes of glaucomatous RGC death^67^. Further validation of Ripa-56's effectiveness is required in additional models, such as high intraocular pressure and genetic mouse models. Secondly, our research confirmed that Ripa-56 effectively alleviated mouse RGC damage induced by glutamate excitotoxicity after intravitreal injection for 3 days. The long-term effects of Ripa-56 need to be observed at extended time points.

## Conclusion

This study demonstrated the efficacy of Ripa-56 in effectively inhibiting glutamate-induced excitotoxicity in R28 cells and NMDA-induced retinal excitotoxicity in mice. This protective effect is likely attributed to the anti-apoptotic, anti-necroptosis, anti-ferroptosis and anti-inflammation properties of Ripa-56. As a result, Ripa-56 shows as a potential retinal protective agent, and further investigation of its therapeutic effects in glaucoma is warranted.

### Supplementary Information

Supplementary Figure 1.

## Data Availability

The datasets used and/or analyzed during the current study are available from the corresponding author on reasonable request.
