# Supplementary figures and images for: Ripa-56 protects retinal ganglion cells in glutamate-induced retinal excitotoxic model of glaucoma
Source: Sci Rep. 2024 Feb 15;14:3834. doi: 10.1038/s41598-024-54075-z (PMC10869350; doi:10.1038/s41598-024-54075-z)

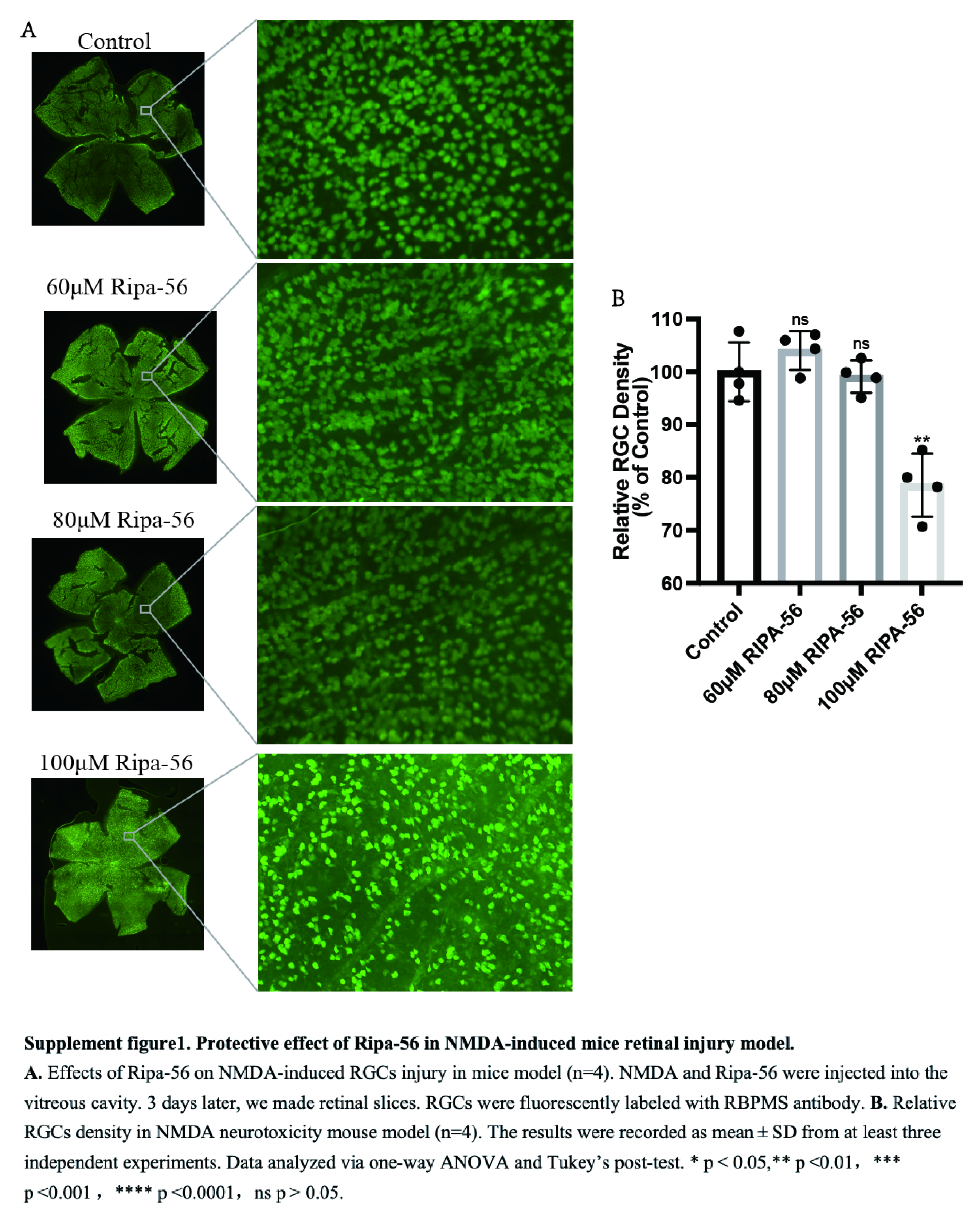

Supplement: Supplementary file 1 — Supplementary Figure 1. [file 41598_2024_54075_MOESM1_ESM.tif]
